# Edible Xanthan/Propolis Coating and Its Effect on Physicochemical, Microbial, and Sensory Quality Indices in Mackerel Tuna (*Euthynnus affinis*) Fillets during Chilled Storage

**DOI:** 10.3390/gels8070405

**Published:** 2022-06-25

**Authors:** Aly Farag El Sheikha, Ayman Younes Allam, Emel Oz, Mohammad Rizwan Khan, Charalampos Proestos, Fatih Oz

**Affiliations:** 1 College of Bioscience and Bioengineering, Jiangxi Agricultural University, 1101 Zhimin Road, Nanchang 330045, China; 2 School of Nutrition Sciences, Faculty of Health Sciences, University of Ottawa, 25 University Private, Ottawa, ON K1N 6N5, Canada; 3 Bioengineering and Technological Research Centre for Edible and Medicinal Fungi, Jiangxi Agricultural University, 1101 Zhimin Road, Nanchang 330045, China; 4 Jiangxi Key Laboratory for Conservation and Utilization of Fungal Resources, Jiangxi Agricultural University, 1101 Zhimin Road, Nanchang 330045, China; 5 Department of Food Science and Technology, Faculty of Agriculture, Minufiya University, Shibin El Kom 32511, Egypt; ayman_alaam@yahoo.com; 6 Department of Food Engineering, Faculty of Agriculture, Ataturk University, Erzurum 25240, Türkiye; emel.oz@atauni.edu.tr (E.O.); fatihoz@atauni.edu.tr (F.O.); 7 Department of Chemistry, College of Science, King Saud University, Riyadh 11451, Saudi Arabia; mrkhan@ksu.edu.sa; 8 Laboratory of Food Chemistry, Department of Chemistry, School of Sciences, National and Kapodistrian University of Athens, 15772 Athens, Greece

**Keywords:** bioactive packaging, xanthan, propolis, physicochemical properties, microbiological analyses, sensory evaluation

## Abstract

Worldwide aquaculture production is increasing, but with this increase comes quality and safety related problems. Hence, there is an urgent need to develop potent technologies to extend the shelf life of fish. Xanthan gum is commonly used in the food industry because of its high-water solubility, stability of its aqueous solutions in a wide pH range, and high viscosity. One of its modern food applications is its use as a gelling agent in edible coatings building. Therefore, in this study, the effect of xanthan coating containing various concentrations (0, 1, 2%; *w*/*v*) of ethanolic extract of propolis (EEP) on physicochemical, microbial, and sensory quality indices in mackerel fillets stored at 2 °C for 20 days was evaluated. The pH, peroxide value, K-value, TVB-N, TBARS, microbiological and sensory characteristics were determined every 5 days over the storage period (20 days). Samples treated with xanthan (XAN) coatings containing 1 and 2% of EEP were shown to have the highest level of physicochemical protection and maximum level of microbial inhibition (*p* < 0.05) compared to uncoated samples (control) over the storage period. Furthermore, the addition of EEP to XAN was more effective in notably preserving (*p* < 0.05) the taste and odor of coated samples compared to control.

## 1. Introduction

With the continuous increase in the world population, which may reach over 9 billion by the year 2050, global food production needs to increase by an estimated 50% at least to keep pace with this population increase and meet its nutritional needs [1]. Fish and fishery products can meet a significant proportion of the world’s food needs by 2050 [2]. In this context, it was noted that the world per capita fish consumption increased from an average of 9.9 kg in the 1960s to 20.5 kg in 2018 [3]. Although fish has high commercial value, it is an extremely perishable product. Mackerel tuna (*Euthynnus affinis*) is a commercially important fish in high demand. As with other fish, Mackerel tuna is easily and rapidly damaged (highly perishable), so it is very susceptible to quality degradation. The major factor causing quality degradation in fish, including mackerel tuna, is microbial activity, even though the first changes are caused by the endogenous enzymes of fish, which ultimately shortens their shelf life [4,5,6]. Microbial deterioration proceeds fast because of the presence of large amounts of low-molecular-weight compounds, high water activity, and high post-mortem (pH > 6) in fish muscles. Hence, cooling is necessary to prolong the shelf life of fish and is often combined with vacuum packaging to prevent the growth of aerobic microflora that cause spoilage [5,7]. Thus, the application of appropriate packaging and/or processing techniques will be the best solution to extend the shelf life of fish and fish products [8]. In addition to the short shelf life, another challenge facing fresh fish consumption is that seafood products take a long time to prepare as meals [9]. Despite freezing and cold storage being significant and frequent methods of preserving fish and fish products, they cannot completely prevent chemical and oxidation reactions in fish and fish products [10]. The reason for the occurrence of these reactions may be related to the presence of polyunsaturated fatty acids in fish and fish products, which oxidize rapidly in the presence of oxygen [11]. Therefore, the use of preservatives, especially natural ones, has become an urgent need to extend the shelf life of perishable foods such as fish [12].

Propolis (bee glue) is a balsamic product obtained from exotic Africanized bees *Apis mellifera* L. [13]. Propolis extract is well-known for its functional properties, such as anti-inflammatory, pharmacological, antiviral, anticancer, antioxidant, antifungal, and antibacterial activities [14]. Natural preservatives benefits have recently been enhanced by incorporating them into various edible coatings and films on food products [15,16]. Due to the edible coatings’ simplicity and eco-friendly nature. Several characteristics distinguish the edible coating. It acts as a carrier for bioactive components and is a semi-permeable barrier to moisture loss, gas exchange, and oxidative reactions [17,18]. Several studies have been conducted showing the possibility of using natural gums such as xanthan in formulating edible coatings and improving their characteristics [19,20,21,22].

Xanthan gum is a polysaccharide produced by *Xanthomonas campestris*, and a food additive that is commonly added to foods as a thickener or stabilizer [23,24]. Xanthan is featured because of its ability to enhance food flavor, consistency, texture, shelf life, and appearance [24]. Xanthan has multiple technological advantages that make it a rich raw material with various applications, especially for food. The features of xanthan could be listed as follows [23,25]: (1) high viscosity at low concentrations: for example, a solution with a concentration of 1% appears almost gel-like at rest, yet pours readily and has a very low resistance to mixing and pumping; (2) high resistance to a wide pH range (2–12) makes xanthan well-suited to foods; (3) high thermal stability; the viscosity is not affected by temperatures in the range of (0–100 °C), and it has excellent freeze-thaw ability; (4) high solubility of xanthan gum renders it appropriate for many applications, including foods; (5) high compatibility with most of the commercially available thickeners.

Furthermore, one of the new food applications of xanthan is its use as a gelling agent in edible coating building [26].

Bioactive edible coatings or films from natural preservatives with antioxidant and antibacterial properties, prolong the shelf life of fish and fish products [27]. The main advantage is that the edible film helps in the reduction of environmental pollution [28,29]. In this sense, there is just one study that indicates the effect of using edible coatings on extending the shelf life of mackerel tuna fish (*Euthynnus affinis*) fillets [30]. Kumar and others’ [30] study aimed to develop a bioactive edible coating from gelatin and chitosan, incorporated with different concentrations of clove oil as a natural preservative, and evaluate their effect on the shelf life of mackerel fillets under refrigerated conditions (4 °C). This edible coating demonstrated its potential as a natural antibacterial agent which can be used for packaging tuna and other fishery products.

Due to the features of propolis as a natural preservative (antioxidant and antimicrobial agent), it could be integrated with xanthan gum in formulating gel-based edible coating [31] to extend the shelf life of fish and fishery products. There is no published data on the application of a xanthan/propolis composite coating to preserve fish and fish products’ quality and shelf life. Therefore, to our knowledge, this is the first paper to study the effects of using a composite edible coating from xanthan containing various levels (0, 1, and 2%) of ethanolic extract of propolis (EEP) on the physicochemical, microbiological, and sensory quality parameters of mackerel tuna fish (*Euthynnus affinis*) fillets during chilled storage (2 °C) for 20 days.

## 2. Results and Discussion

### 2.1. Test Probabilities for Physicochemical, Microbiological, and Sensory Criteria of Mackerel Tuna Fillets—Multi-Aspect Variance Analysis, including Interactions

The storage time and coating treatment of mackerel tuna fillets can have a significant impact on their physicochemical, microbiological, and sensory quality indices. The data presented in Table 1 show a significant effect (*p* < 0.001) of storage period on all measured parameters except taste, in which the significant effect was *p* < 0.01. Furthermore, Table 1 illustrates a significant effect (*p* < 0.001) of coating treatment on all measured parameters except TBARS, TVC, PTC, and Enterobacteriaceae, in which the significant effect was *p* < 0.01. In addition, the coating treatment had a smaller effect on K-value (*p* < 0.05). The interactions between the storage time and the coating treatment were also indicated for all the tested parameters. The storage time and coating treatment had a significant effect (*p* < 0.001) on all measured parameters.

### 2.2. Physicochemical Analyses of Mackerel Tuna Fillets

#### 2.2.1. pH Values of Mackerel Fillets

**Figure 1:**
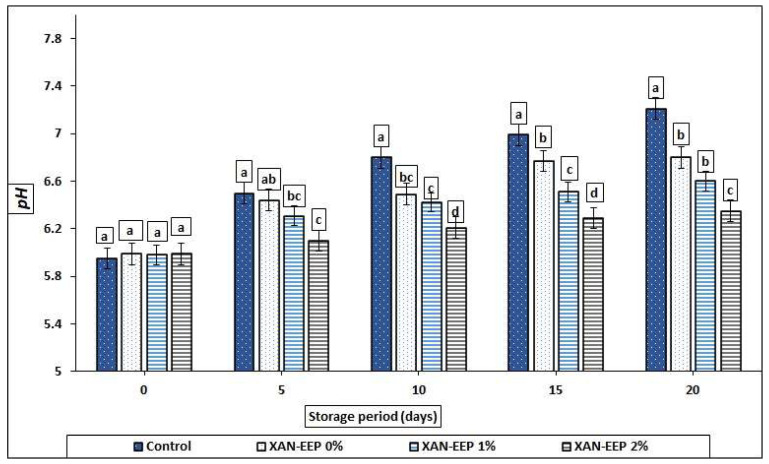
The influence of coating treatments on pH values in mackerel tuna fillet samples during storage at 2 °C for 20 days. Control: Uncoated mackerel tuna fillet samples (soaked samples in sterile distilled water). XAN-EEP 0%: Coated samples with xanthan containing (0%) ethanolic extract of propolis. XAN-EEP 1%: Coated samples with xanthan containing (1%) ethanolic extract of propolis. XAN-EEP 2%: Coated samples with xanthan containing (2%) ethanolic extract of propolis. ^a–d^: Within a column, different superscripts indicate significant differences (*p* < 0.05).

Figure 1 shows the pH value changes in mackerel tuna fish fillets stored at 2 °C for 20 days. The primary pH values of fresh mackerel tuna fillets (pH 5.93–5.98) were consistent with previous studies [32,33]. In our study, the pH value of control samples increased from 5.95 to 7.21 after 20 days of cold storage, while the pH values for XAN-EEP 0%, XAN-EEP 1%, and XAN-EEP 2% samples after 20 days of storage were 6.81, 6.60, and 6.35, respectively. These results exhibited the protective effect of XAN edible coating against spoilage, which was significantly (*p* < 0.05) increased by propolis, especially in the higher dose group. The lower pH value of the other treatments (XAN-EEP 0%, XAN-EEP 1%, and XAN-EEP 2%) could have prevented exogenous (microbial) and endogenous proteases from acting in treated mackerel tuna fillets through the storage period. Propolis’ antimicrobial and antioxidant properties may be responsible for the observed pH changes in stored fish fillets, preventing changes in proteolysis and microbiological development [34].

Furthermore, the pH values of coated and uncoated mackerel tuna fillets increased as the storage period increased. At the end of the storage period, the increase in pH values of the uncoated samples (controls) was more pronounced. This can happen as a result of the accumulation of ammonia and amino acid degradation products, which causes the pH to rise [33]. An increase in the pH values of stored fish may be linked to the production of peptides, amino acids, and ammonia due to increased protease activity or microbial development [35,36].

#### 2.2.2. Oxidative Stability of Mackerel Tuna Fillets

The deterioration of the quality of fish and its products during storage is mainly due to the oxidation of lipid [11]. Lipid peroxidation is the reaction of oxygen with unsaturated lipids; hence, one of the methods that delays or prevents oxidation processes is the use of edible coatings for fish fillets [37] because the edible coatings can guarantee performance as a low oxygen barrier [24].

**Figure 2:**
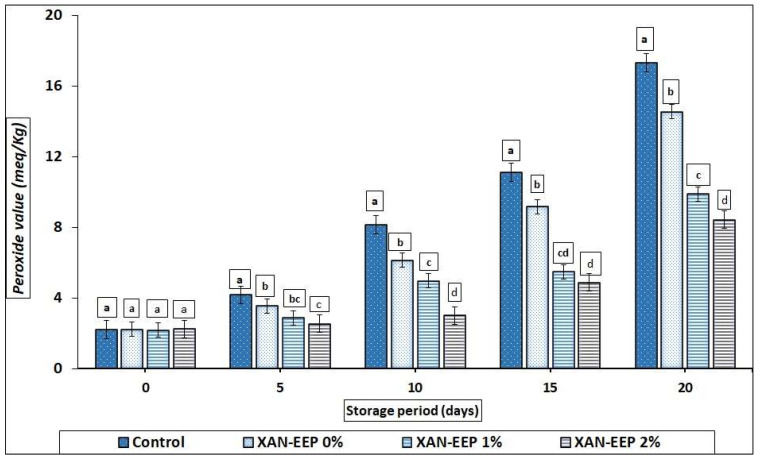
The influence of coating treatments on peroxide values (meq/kg) in mackerel tuna fillet samples during storage at 2 °C for 20 days. Control: Uncoated mackerel tuna fillet samples (soaked samples in sterile distilled water). XAN-EEP 0%: Coated samples with xanthan containing (0%) ethanolic extract of propolis. XAN-EEP 1%: Coated samples with xanthan containing (1%) ethanolic extract of propolis. XAN-EEP 2%: Coated samples with xanthan containing (2%) ethanolic extract of propolis. ^a–d^: Within a column, different superscripts indicate significant differences (*p* < 0.05).

The peroxide value (POV) is a substantial indicator of fat rancidity, but how does fat rancidity happen? Rancidity happens through the process of lipid oxidation, which is accompanied by the production of free radicals, which in turn leads to the formation of aldehydes and ketones, all of which, of course, negatively affect the quality of fish [38]. As the storage period progressed, the peroxide values in coated and uncoated mackerel tuna fillets increased, with the control (uncoated) samples having the highest (*p* < 0.05) peroxide value at each interval storage period (Figure 2). The peroxide value of the control (uncoated fillet samples) increased from 2.22 to 17.32 meq peroxides/kg lipid, while during this time, the peroxide value of XAN-EEP 0%, XAN-EEP 1%, and XAN-EEP 2% increased from 2.23 to 14.55, 2.18 to 9.89, and 2.25 to 8.44 meq peroxides/kg lipid, respectively, after 20 days of chilled storage. In all treatments (coated fillet samples), the values were significantly reduced (*p* < 0.05) compared to the control samples. In this context, Roy et al. [39] found that the composite coating based on propolis could reduce the peroxide index in coated meat products over the storage period compared to the control (uncoated samples). The XAN-EEP 2% treatment resulted in a maximal decrease in the peroxide formation, followed by XAN-EEP 1% and XAN-EEP 0%; this condition may be due to the potent antioxidant activity of XAN-EEP 2%. These results may be in line with what was mentioned by Shavisi et al. [40]. They observed that a polylactic acid (PLA) film containing ethanolic extract of propolis (EEP) reduced the peroxide value of minced beef more than the control samples that were stored in the refrigerator.

**Figure 3:**
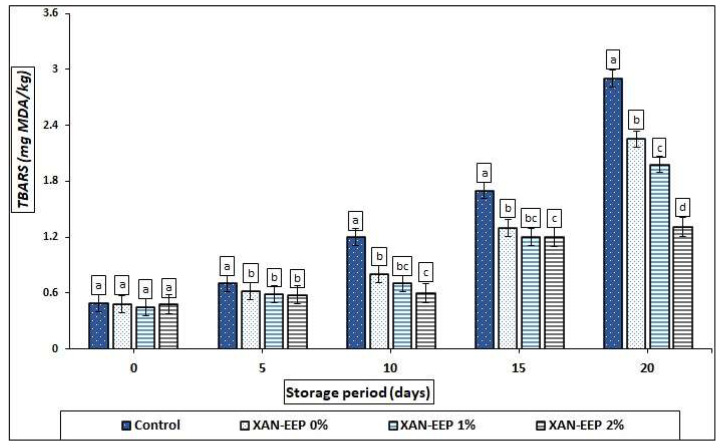
The influence of coating treatments on TBARS values (MDA mg/kg) in mackerel tuna fillet samples during storage at 2 °C for 20 days. Control: Uncoated mackerel tuna fillet samples (soaked samples in sterile distilled water). XAN-EEP 0%: Coated samples with xanthan containing (0%) ethanolic extract of propolis. XAN-EEP 1%: Coated samples with xanthan containing (1%) ethanolic extract of propolis. XAN-EEP 2%: Coated samples with xanthan containing (2%) ethanolic extract of propolis. ^a–d^: Within a column, different superscripts indicate significant differences (*p* < 0.05).

The results for TBARS which is an indicator of lipid oxidation [41] of mackerel tuna fillets coated in (Figure 3) showed a significant effect of the coating on the oxidation of mackerel fillets. During refrigeration, the TBARS values of XAN-EEP 0%, XAN-EEP 1%, and XAN-EEP 2% were significantly (*p* < 0.05) lower than the control. After 20 days of cold storage, the TBARS values of the XAN-EEP 0%, XAN-EEP 1%, and XAN-EEP 2% treatments were 2.25, 1.98, and 1.31 mg MDA/kg, respectively. The malondialdehyde (MDA) levels in the treated fillets (XAN, XAN-EEP 1%, and XAN-EEP 2%) were significantly lower (*p* < 0.05) than in the control sample (2.9 mg MDA/kg). Connell [42] also mentioned that the acceptable limit for the value of TBARS in a fish sample is in the range of 1 to 2 mg MDA/kg, and if the value exceeds this limit, an unpleasant smell of fish begins to develop. All tested samples for all treatments in our study exceeded the TBARS value limit after 20 days of storage.

TBARS values were significantly lower in the EEP-containing coated mackerel tuna fillet samples than in others, most likely due to the antioxidants present (EEP) [40]. Additionally, the highest effects were noted with XAN-EEP at a concentration of 2%.

#### 2.2.3. Total Volatile Basic Nitrogen (TVB-N) of Mackerel Tuna Fillet Samples

**Figure 4:**
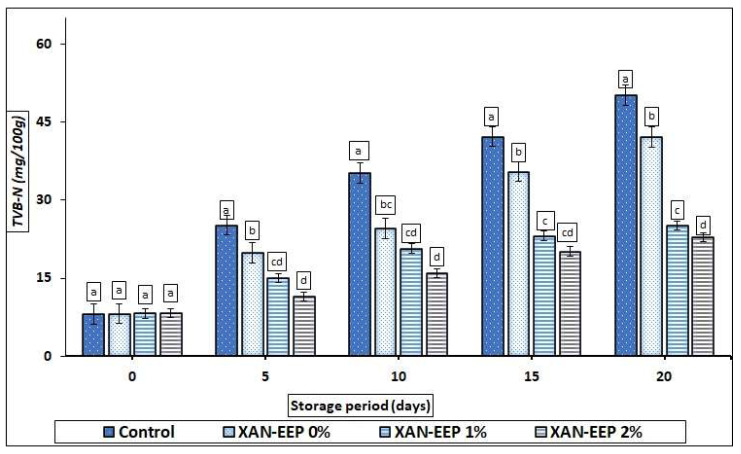
The influence of coating treatments on TVB-N values (mg/100 g) in mackerel tuna fillet samples during storage at 2 °C for 20 days. Control: Uncoated mackerel tuna fillet samples (soaked samples in sterile distilled water). XAN-EEP 0%: Coated samples with xanthan containing (0%) ethanolic extract of propolis. XAN-EEP 1%: Coated samples with xanthan containing (1%) ethanolic extract of propolis. XAN-EEP 2%: Coated samples with xanthan containing (2%) ethanolic extract of propolis. ^a–d^: Within a column, different superscripts indicate significant differences (*p* < 0.05).

Amongst the important indicators of spoilage is TVB-N, which results from the degradation of proteins and non-protein nitrogen compounds as a response to bacterial activity as well as the presence of endogenous enzymes [43]. Figure 4 shows the changes in TVB-N values for all chip processors during cryogenic storage. At the start of storage (zero-time), TVB-N content ranged from 8.12 to 8.20 mg N/100 g for all mackerel tuna fillet samples. Over time TVB-N values increased for all samples, which was, of course, consistent with increases in pH values during later stages of storage. The results of our study are in line with those obtained by Yu et al. [44]. On the 20th day, TVB-N values for the control, XAN-EEP 0%, XAN-EEP 1%, and XAN-EEP 2% were 50.19, 42.15, 27.14, and 22.87 mg N/100 g, respectively. Thirty-five to forty milligrams of nitrogen per one hundred grams is the acceptable limit for TVB-N values in fresh fish, as reported by Connell [42]. Grigorakis et al. [45] suggested that the acceptable limit for TVB-N values in chilled sea bass is 19–20 mg N/100 g. Twenty-five to thirty-five of nitrogen per one hundred grams was considered a limit for mackerel tuna fillet damage in our study. The edible films may extend the shelf life of the fish fillet by reducing gas permeability and penetration, especially oxygen permeability, thereby limiting bacterial growth and activity [46].

In comparison to the other treatments, the propolis-treated mackerel tuna fillets had the lowest TVB-N values. Similar observations were obtained by Bazargani-Gilani et al. [47]. These results can be interpreted based on the ability of propolis to inhibit microbial activity, including the inhibition of bacteria responsible for the deamination reaction of non-protein nitrogen (NPN) components [46].

#### 2.2.4. *K*-Value of Mackerel Tuna Fillet Samples

**Figure 5:**
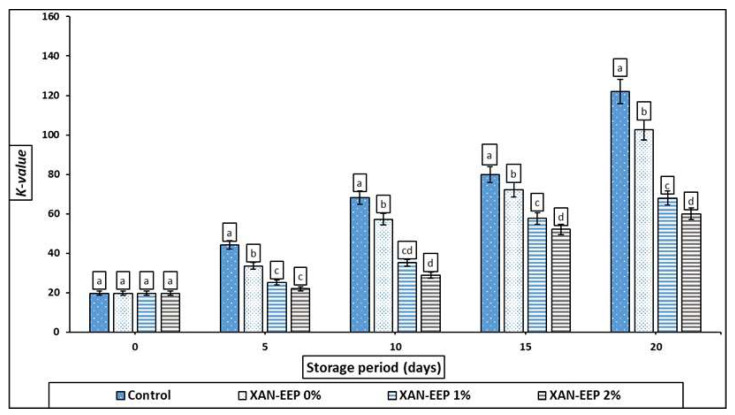
The influence of coating treatments on K values (%) in mackerel tuna fillet samples during storage at 2 °C for 20 days. Control: Uncoated mackerel tuna fillet samples (soaked samples in sterile distilled water). XAN-EEP 0%: Coated samples with xanthan containing (0%) ethanolic extract of propolis. XAN-EEP 1%: Coated samples with xanthan containing (1%) ethanolic extract of propolis. XAN-EEP 2%: Coated samples with xanthan containing (2%) ethanolic extract of propolis. ^a–d^: Within a column, different superscripts indicate significant differences (*p* < 0.05).

Endogenous biochemical changes occur in fish muscle during postmortem fish storage, among which is nucleotide degradation [48]. Calculation of the contents of ATP and its associated degradation products is an effective indicator for monitoring the freshness of fish fillets [47]. Changes in the K value during cryogenic storage of mackerel tuna fillets are shown in Figure 5. The initial K-values of the control and treated mackerel tuna samples ranged from 15.31 to 16.82%. K-values of uncoated (control) and coated mackerel tuna fillet samples increased significantly (*p* < 0.05) with storage time. Additionally, the treatments illustrated significantly lower K-values (*p* < 0.05) than the control sample. According to previous studies, the rejection level of the K-value was close to 60% [49]. Control exceeded this limit on the 10th day (68.14%), while XAN-EEP 0% exceeded this limit on the 15th day (72.19%), and both XAN-EEP 1% and XAN-EEP 2% exceeded this limit on the 20th day (68.04, 59.99%).

### 2.3. Microbiological Analyses of Mackerel Tuna Fillets

Generally, propolis’ antimicrobial characteristics may be responsible for the inhibition of microbial growth in stored fish fillets [50]. Specifically, the following sections will focus on the changes in each microbial group in mackerel tuna fillet samples.

#### 2.3.1. Total Viable Count (TVC)

**Figure 6:**
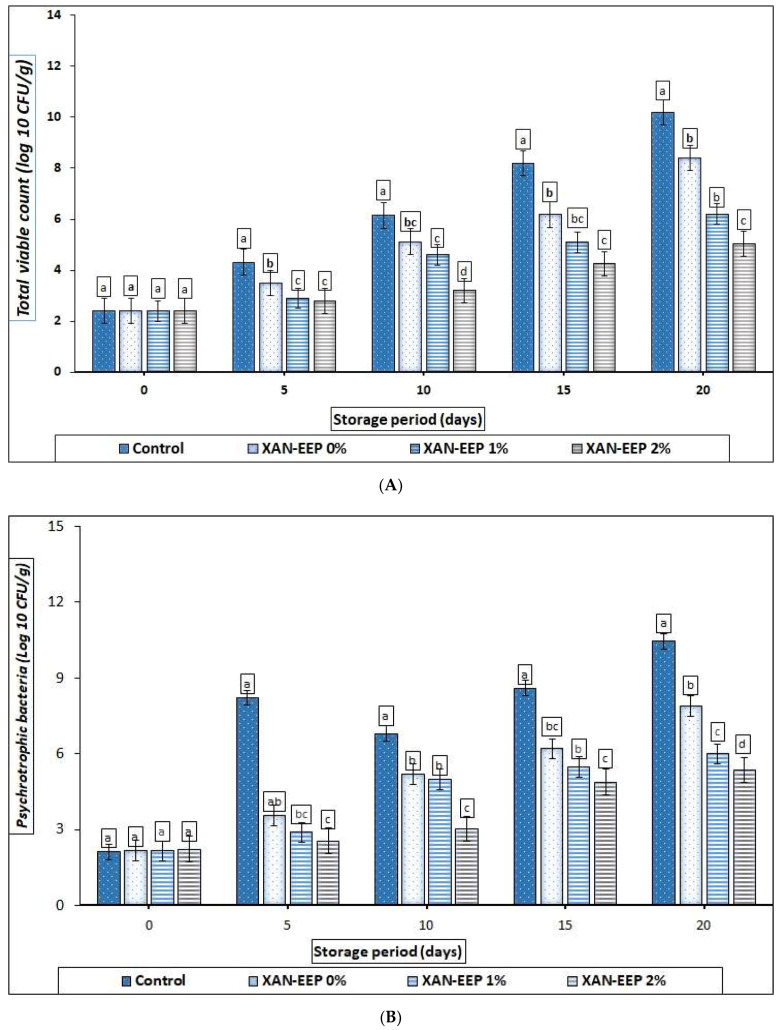

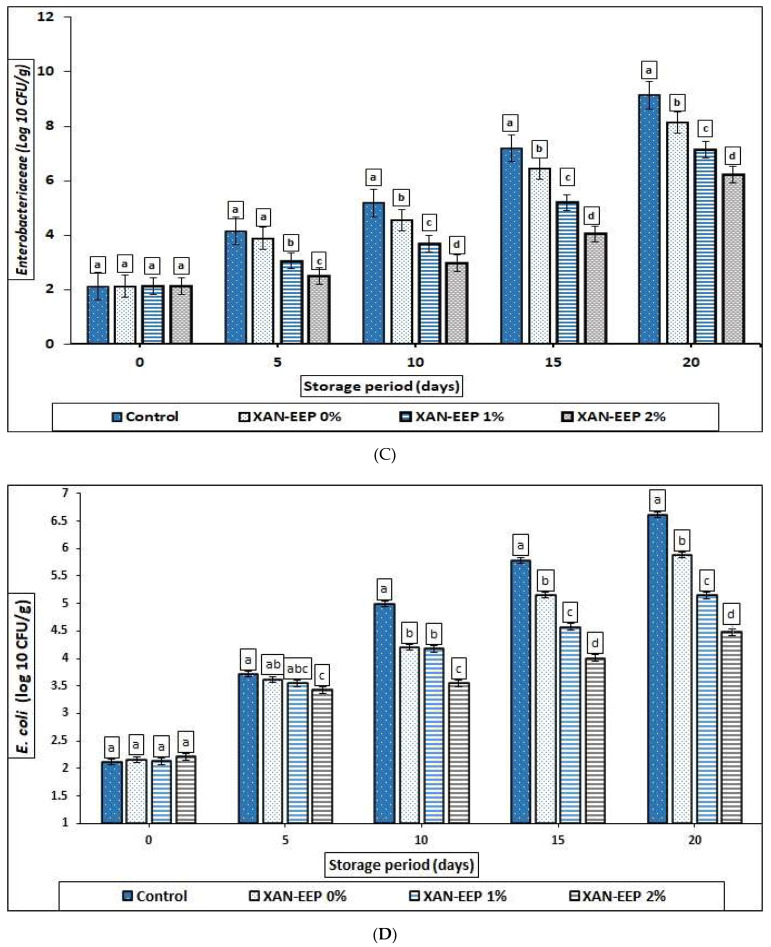

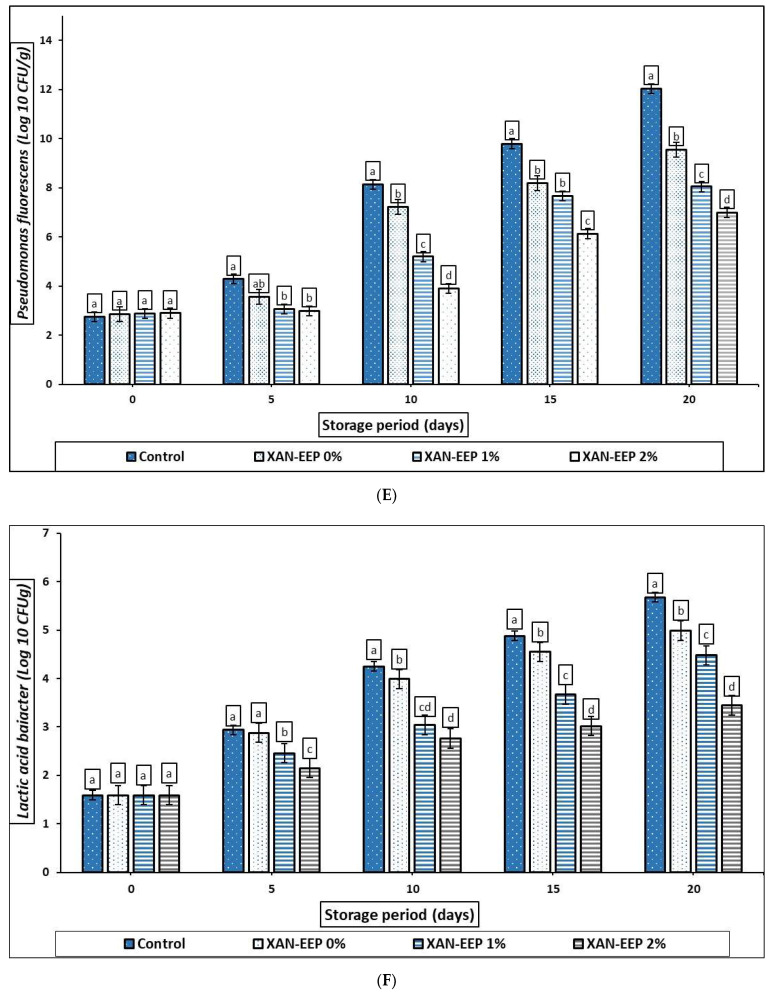

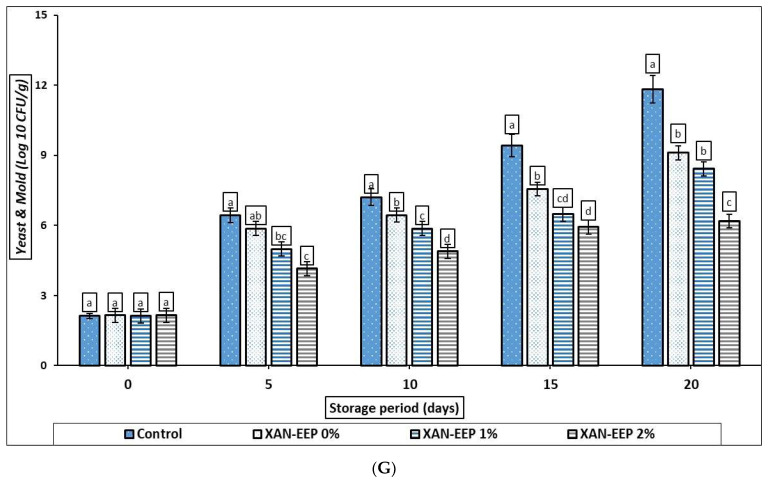
The influence of coating treatments on (**A**) total viable count (TVC) (log_10_ CFU/g), (**B**) psychotropic count (PTC) (log_10_ CFU/g), (**C**) Enterobacteriaceae (log_10_ CFU/g), (**D**) E. coli (log10 CFU/g), (**E**) *Pseudomonas fluorescens* (log10 CFU/g), (**F**) lactic acid bacteria (LAB) (log_10_ CFU/g), and (**G**) yeasts and molds (log_10_ CFU/g) in mackerel tuna fillet samples during storage at 2 °C for 20 days. Control: Uncoated mackerel tuna fillet samples (soaked samples in sterile distilled water). XAN-EEP 0%: Coated samples with xanthan containing (0%) ethanolic extract of propolis. XAN-EEP 1%: Coated samples with xanthan containing (1%) ethanolic extract of propolis. XAN-EEP 2%: Coated samples with xanthan containing (2%) ethanolic extract of propolis. ^a–d^: Within a column, different superscripts indicate significant differences (*p* < 0.05).

Changes in total viable count (TVC) of mackerel tuna fillet samples during refrigerated storage are shown in Figure 6A. The initial TVC (log_10_ CFU/g) of all samples, including the control and treatments, ranged from 2.5 to 3.0. Compared to the values reported by Yu et al. [44] for grass carp fillets (4.90 log_10_ CFU/g), the values obtained in our study were lower. The reason for this may be attributed to individual differences or the handling of the fish during processing. The lower initial TVC for coated mackerel tuna fillets indicated that XAN-EPP coating reduced the microbial population. According to the International Commission on Microbiological Specifications for Foods (ICMSF) [51], the maximum allowable TVC is 7.0 log_10_ CFU/g. Based on that and looking at the results of our study, it was found that over the storage period, there was a noticeable increase (*p* < 0.05) in TVC for untreated samples compared to treated samples, until the untreated samples (control group) exceeded the permissible limit of TVC after 11 days. XAN-EEP 0% samples have exceeded the TVC limit after 16 days. For mackerel tuna fillets treated with 1% and 2% ethanolic extract of propolis (EEP), TVC was below the limit level during the whole storage period.

#### 2.3.2. Psychotropic Count (PTC)

Among the major pathogens of microbial spoilage of refrigerated fish fillets are psychotropic bacteria [45]. Changes in the PTC of fish fillets are shown in Figure 6B. PTC of mackerel tuna fillets increased progressively (*p* < 0.05) from initial values of 2.11, 2.17, 2.15, and 2.22 log_10_ CFU/g to final values of 10.44, 7.88, 5.99, and 5.34 log_10_ CFU/g for control, XAN-EEP 0%, XAN-EEP 1%, and XAN-EEP 2%, respectively. The results depicted that the composite edible coating formed from xanthan and ethanolic extracted propolis significantly (*p* < 0.05) inhibited the growth of the total psychotropic bacteria.

#### 2.3.3. Enterobacteriaceae

Based on previous studies, Enterobacteriaceae were found to be among the main spoilers in rainbow trout fillets stored at 4 °C [52]. The initial count of Enterobacteriaceae was 2.12 log_10_ CFU/g on trout fillets coated under fridge temperature [47,53]. After 15 storage days, Enterobacteriaceae counts reached 4.04, 5.19, 6.44, and 7.18 log_10_ CFU/g in the XAN-EEP 2%, XAN-EEP 1%, XAN-EEP 0%, and control fillets, respectively (Figure 6C). Moreover, according to the studies done by Volpe et al. [53], Bazargani-Gilani et al. [44], steady growth in Enterobacteriaceae was observed for refrigerator-stored trout chips. The findings of these studies are consistent with what was revealed by our study of the ability of coating with XAN and EEP to reduce the growth rate of Enterobacteriaceae in fillets (*p* < 0.05) compared to the uncoated samples (control samples) during cold storage. The lowest number of Enterobacteriaceae communities was found in XAN-EEP 2% fillet samples, followed by XAN-EEP 1% and XAN-EEP 0%.

As mentioned by Jalali et al. [54], *Escherichia coli* O157:H7 is the major member of Enterobacteriaceae found in the chilled silver carp flesh. The initial count of *E*. *coli* O157:H7 ranged from 2.11 to 2.21 log_10_ CFU/g. After 20 storage days, *E*. *coli* O157:H7 counts reached 4.48, 5.15, 5.88, and 6.61 log_10_ CFU/g in the XAN-EEP 2%, XAN-EEP 1%, XAN-EEP 0%, and control fillets, respectively (Figure 6D). A previous study showed that propolis extracts can be considered natural preservatives. Their efficacy has been proven to inhibit *Escherichia coli* bacteria in vitro due to the polyphenol compounds that propolis extracts contain, which are known for their antimicrobial effect. Among these phenolic compounds is gallic acid, known for its antibacterial activity [55]. Phenolic compounds act on the bacterial cell membrane, interfere with nucleic acid synthesis, inhibit bacterial metabolism, coagulate cytoplasmic proteins, and interfere with biofilm formation [56].

#### 2.3.4. Pseudomonas Fluorescens

According to the primary count of about 2.74–2.89 log_10_ CFU/g for *Pseudomonas fluorescens* (day 0) of the mackerel tuna fillet samples (Figure 6E), similar initial numeration (day 0) related to the rainbow trout was also found by other studies [44]. During the storage time, the *P*. *fluorescens* count rose to the final numeration of 12.03 log_10_ CFU/g (control fillets), while the counts of XAN-EEP 0%, XAN-EEP 1%, and XAN-EEP 2% reached 9.55, 8.04, and 6.99 log_10_ CFU/g at the last interval, being less than the control fillets. The *P. fluorescens* count in all groups was significantly (*p* < 0.05) less than the control, showing that EEP-containing treatments were the strongest concerning the inhibition treatments of *P*. *fluorescens*. The use of propolis extracts can be effective in inhibiting the activity of *P*. *aeruginosa* that causes chronic putrefaction as shown by studies conducted by Mohammadzadeh et al. [57], and De Marco et al. [58].

#### 2.3.5. Lactic Acid Bacteria

Lactic acid bacteria (LAB) as facultative anaerobic bacteria are part of the original microflora of mackerel tuna flesh; hence, the number of these bacteria can increase under both aerobic and anaerobic conditions [59]. As shown in Figure 6F, it is clear that the initial number of LAB was 1.59 log_10_ CFU/g and did not exceed 4.88 log_10_ CFU/g in control fillets until the 15th day of the storage period. It was also observed that the LAB counts of the XAN and XAN-EEP fillet samples were significantly lower (*p* < 0.05) compared to the control samples (uncoated fillets) during the refrigerated storage period.

The best treatment in terms of inhibiting LAB proliferation in mackerel tuna fillet samples among the other tested groups was XAN-EEP 2% compared to the other tested groups, and this could be attributed to the synergistic antimicrobial effect of EEP. It has been known that LAB bacteria are the most resistant Gram-positive bacteria to antimicrobial agents [60]. The results of our study confirmed this as LAB bacteria were more resistant compared with other spoilage bacteria versus XAN combined with EEP.

This conclusion regarding the synergistic antimicrobial effect of EEP may agree with what was indicated by Duman and Özpolat [61] concerning the effect of aqueous extract of propolis during storage of shibuta (*Barbus grypus*) fillets at 4 °C. It was observed that 0.5% aqueous extract of propolis significantly reduced the number of shibuta’s lactic acid bacteria at all storage times compared to the control samples. Duman and Özpolat [61] attributed this effect to the phenolic content of propolis.

In another study conducted in Greece and Cyprus on evaluating the antibacterial activities of propolis ethanolic extracts (PEs), the results concluded that the minimum inhibitory concentration (MIC) of all studied propolis ethanolic extracts was higher for lactic acid bacteria compared to the other tested bacterial species (*Listeria monocytogenes*, *Staphylococcus aureus*, and *Bacillus cereus*) [62].

#### 2.3.6. Yeasts/Molds

The yeast and mold species are common agents of microbial spoilage in refrigerated fish [63]. A in prior investigations, the primary count (day 0) of yeast/mold of mackerel tuna fillets was 2.13–2.16 log_10_ CFU/g (Figure 6G) [44,64]. It was shown from the results of all treatments (XAN-EEP 0%, XAN-EEP 1%, and XAN-EEP 2%) in the present study that they had a significant ability (*p* < 0.05) to reduce the number of yeasts/molds compared to the untreated fillet samples (control) under cooling conditions (Figure 6G). These results were in agreement with Duman and Özpolat [61], confirming the antifungal activity of propolis from refrigerated shibuta fillets.

Other studies have highlighted the role of propolis extracts against *Candida tropicalis* and *Candida albicans* [62], and also against *Aspergillus niger* and *Candida albicans* [57].

### 2.4. Sensory Evaluation

**Figure 7:**
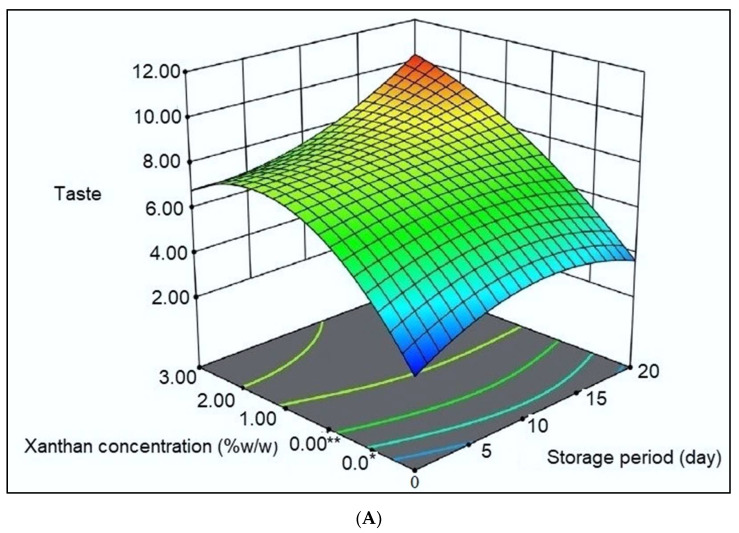

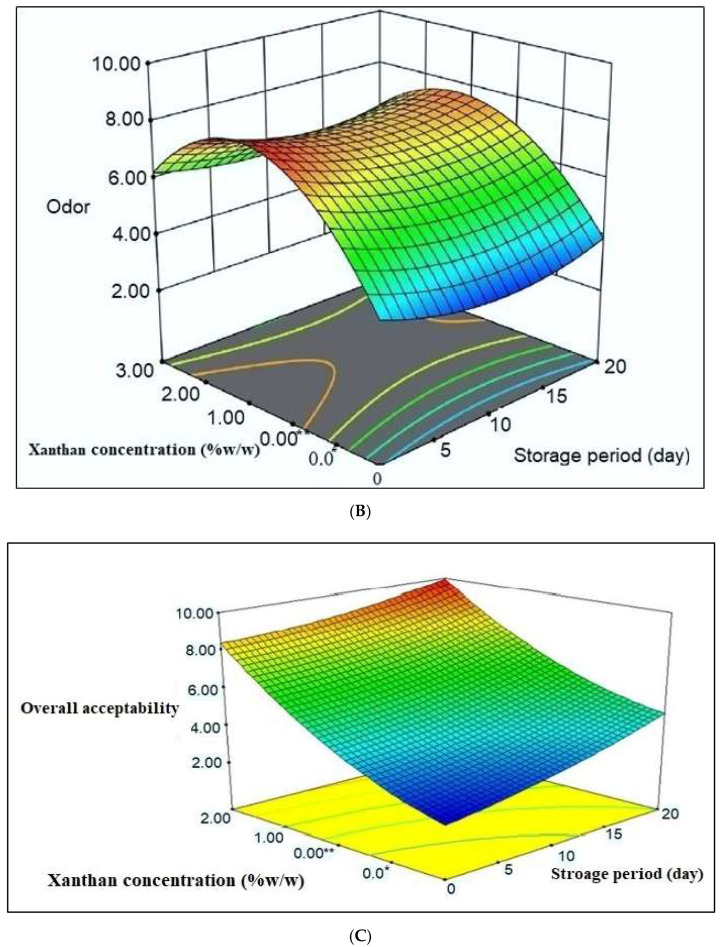
The response surface plot of coating treatments on the (**A**) taste, (**B**) odor, and (**C**) overall acceptability of mackerel tuna fillet samples during storage at 2 °C for 20 days. 0.0 *: Control samples [Uncoated mackerel tuna fillet samples (soaked samples in sterile distilled water)]. 0.00 **: XAN-EEP 0% [Coated samples with xanthan containing (0%) ethanolic extract of propolis]. 1.00: XAN-EEP 1% [Coated samples with xanthan containing (1%) ethanolic extract of propolis]. 2.00: XAN-EEP 2% [Coated samples with xanthan containing (2%) ethanolic extract of propolis].

It is worth noting that the use of xanthan and ethanolic extract of propolis as food-grade components in coating-forming makes it safe for consumers [65,66]. The freshness of mackerel tuna fillets during storage was assessed sensorially by taste, odor, and overall acceptability. At the beginning of the storage period, all groups of fillets were characterized by a smell of fresh fish and a distinctive shiny surface, but with the continuation of the cold storage process, the sensory properties of the samples deteriorated, but the rate of deterioration was significantly faster (*p* < 0.05) in the uncoated fillet samples (control) compared to the samples coated with xanthan/ethanolic extract of propolis (Figure 7A–C; Supplementary Table S1). According to Bazargani-Gilani and Pajohi-Alamoti [67], the permissible sensory level must be higher than 4 for the samples of fish fillets to be fit for consumption. Fishy and putrid odors increased gradually in control after 10 days of storage. Microbial damage and the consequent accumulation of receptors, such as trimethylamine (TMA) and biogenic amines, are the cause of unpleasant odors [68]. The results obtained in our study from the panelists in terms of the general acceptance of all the fillet samples under examination showed that: (a) uncoated fillet samples (control) had a shelf life of fewer than 11 days; (b) treatment with XAN-EEP 0% had a viability of more than 11 days; (c) treatment with XAN-EEP 1%, viable for 15 days; (d) treatment with XAN-EEP 2% had a viability of 20 days.

These results can be attributed to the fact that the incorporation of EEP into the XAN coating significantly (*p* < 0.05) preserved the general acceptability scores and the fresh organoleptic characteristics of taste and aroma in trout meat until the last period of time. These results are also in agreement with what was reported by Duman and Özpolat [61] about shibuta fillets.

The results of general acceptance of the studied treatments can be linked to the antimicrobial effect of EEP associated with its content of phenolic compounds [47].

## 3. Conclusions

Through the results of physical, chemical, microbiological, and sensory analyses conducted in our study, it can be concluded that the composite coating of xanthan and ethanolic extract of propolis preserves the properties of mackerel tuna fillets for a longer time when stored under refrigerated conditions. For example, the shelf life of mackerel tuna steaks extended by about 4 and 7 days for XAN-EEP 1% and XAN-EEP 2%, respectively, compared to the uncoated mackerel tuna fillet samples (control). Xanthan gum is a natural gelling agent that has advantages over synthetic ones owing to its safer, biodegradable nature. Moreover, the orientation of the food coating industry toward these naturally derived gelling agents has led to increasing efforts to discover, extract, and purify such compounds from the natural origin.

This work presented an edible coating containing propolis as a potent, natural, safer, and cost-effective alternative to synthetic preservatives to produce active packaging coatings that might be applicable for other food types. However, further studies are required to identify the effect of propolis on other fish species and fishery products. Furthermore, more applications of EEP-containing packages or coatings on new food models should be tested in future works.

## 4. Materials and Methods

### 4.1. Materials

Propolis was taken from an apiary around Shibin El-Kom City, Menoufia, Egypt, and kept frozen (−18 °C) until used. A total number of 40 samples of mackerel tuna fish (*Euthynnus affinis*) with an average weight of 100–150 g were obtained from a local aquacultural farm (Sadat City, Egypt). Mackerel tuna fish samples were transported to the laboratory in insulated boxes containing ice within one hour of fishing. All experiments were performed in April 2021 at the Department of Food Science and Technology, Faculty of Agriculture, Menofiya University, Shebin El-Kom City, Egypt.

### 4.2. Chemicals and Reagents

Xanthan gum with a molecular weight of around 500 kDa, glycerol, ethanol, methanol, potato dextrose agar, peptone, nutrient agar, plate count agar, and chemical reagents were obtained from Sigma-Aldrich (St. Louis, MO, USA). Trichloroacetic acid (TCA) and thiobarbituric acid (TBA) were procured from Sigma-Aldrich Chemie GmbH (Eschenstr, Taufkirchen, Germany). Stomacher was obtained from Lab Blender 400 (London, UK). Violet red bile glucose agar (VRBGA) was obtained from Trafalgar Scientific Ltd. (Leicester, UK). Potato dextrose agar (PDA) was purchased from Biokar diagnostics (Allonne, France). Man rogosa sharpe agar (MRS) was purchased from Oxoid Ltd. (Basingstoke, UK). Sorbitol-MacConkey agar (SMAC) was obtained from Merck (Darmstadt, Germany).

### 4.3. Preparation of Ethanolic Extract of Propolis (EEP)

The frozen crude propolis kept at –18 °C was grinded in a mortar until a powder was obtained, then blended with ethanol at a ratio of 25:100 g/mL, stirred at 500 rpm for 24–30 h using an Earlene shaker. The resulting solution was then filtered and evaporated at 40–45 °C using a rotary evaporator (Rotavapor RE121, Büchi, Fawil, Switzerland). Lastly, the concentrated extracts were dried at 50–55 °C in a vacuum oven. The final ethanolic extract of propolis (EEP) was stored at −18 °C until used.

### 4.4. Preparation of Coating Formulas

The coating solution was prepared using sterile distilled water and 1.5% xanthan (*w*/*v*). As a plasticizer agent, glycerol 0.5% (*v*/*v*) was utilized in the xanthan (XAN) coating solution, followed by stirring for 30 min on magnetic stirrer. The gel formed after heating the coating solution at 85–90 °C for 3–5 min, then the solution was cooled at room temperature. Different percentages (0, 1, and 2%) of ethanolic extract of propolis (EEP) were added to xanthan to prepare the composite coating formulas (XAN-EEP 0%, XAN -EEP 1%, and XAN-EEP 2%).

### 4.5. Dip Coating Procedure

There were 48 fish divided into four groups (each 12 fish). The mackerel fillet samples were skinned and washed with sterile water. Then the fillets were cut into portions of approximately 3.5 cm × 2.5 cm × 1.6 cm (10–15 g). The mackerel fillet samples were divided into 4 groups. The first group was soaked in sterile distilled water to prepare the control samples (uncoated). The other three groups were soaked in the XAN/EEP composite coating solution for 2 min to prepare the three coated treatments (XAN-EEP 0%, XAN-EEP 1%, and XAN-EEP 2%). The mackerel fillet samples were dipped in respective coating solutions (XAN-EEP 0%, XAN-EEP 1%, and XAN-EEP 2%) in a ratio of 1:2 (*w*/*v*) for 5 min and were then air dried on filter paper for 15–20 min. After that, each sample for each treatment was placed individually in a sterile zip plastic stomach bag under aerobic conditions and all packages were stored in the refrigerator at a temperature of 2 °C. Physical, chemical, microbial, and sensory analyses were carried out every 5 days, starting from 0 to over 20 days from the storage process.

### 4.6. Physicochemical Quality Criteria Analyses

#### 4.6.1. Analysis of pH Value

Ten grams of each sample of fish fillets from all treatments were placed in 100 mL of distilled water and then the homogenization process was carried out for about 30 s; the pH-meter (350 Jenway pH meter, Fisher Scientific, Leicestershire, UK) was calibrated with the pH 7 buffer solution and pH 4 buffer solution, then the electrode was rinsed with distilled water and wipe with a lint-free tissue. After that, the electrode was submerged into each prepared sample to measure its pH value [69].

#### 4.6.2. Oxidative Stability of Mackerel Fillets

Peroxide and TBARS values were used to determine the oxidative stability of mackerel fillets.

1. Determination of peroxide value

By adopting the procedure described by Shon and Chin [70], the peroxide value of mackerel fillets was calculated. Five grams of fillet sample was heated in a water bath at 60 °C for 3 min, followed by the addition of 30 mL of a solution of acetic acid-chloroform (3:2 *v*/*v*) accompanied by thorough mixing by stirring to ensure homogeneity of the sample and also to dissolve the fat. Then a filtration process was carried out, followed by the addition of 0.5 mL of saturated potassium iodide solution to the filtrate. Titration was carried out with a standard solution of sodium thiosulfate (25 g/L) in the presence of a starch solution as an indicator. The value of peroxide was expressed in peroxide equivalent units, in milliequivalent peroxides per kilogram of lipid, which was calculated by the following equation:

(1) $$POV\;\left(\mathrm{meq}.{\mathrm{kg}}^{-1}\right)=\frac{S\times N}{W}\times 1000$$

where *S* is the volume of titration (mL), *N* is the normality of the sodium thiosulfate solution, and *W* is sample weight (kg).

2. Determination of TBARS (Thiobarbituric Acid-Reactive Substances)

Each fish fillet sample (5 g) was dispersed in 20 mL of thiobarbituric acid solution (0.375% thiobarbituric acid, 15% trichloroacetic acid, and 0.25 mol/L HCl). The mixture was heated in boiling water for 10 min, cooled with water, and centrifuged at 3600× *g* for 20 min at room temperature. Then, the TBARS value of the coated fish fillets was assessed spectrophotometrically at 531 nm by spectrophotometer (Model UV-VIS- 2802PC, USA) according to the method described by Song et al. [49]. TBARS values were expressed in milligrams of malondialdehyde (MDA) per kilogram of fish fillet.

#### 4.6.3. Total Volatile Basic Nitrogen (TVB-N) Measurement

Total volatile basic nitrogen (TVB-N) was measured in a mackerel meat sample (10 g) according to the method described by Sallam et al. [71], where the sample was dispersed in 100 mL of distilled water by stirring for 30 min and then filtered. Then, to 5 mL of the filtrate, 5 mL of a MgO solution (1%) was added and a Kjeldahl apparatus was used to distill the sample. The results were calculated as milligrams of nitrogen (N) per 100 g of fish fillets.

#### 4.6.4. *K*-Value Determination

The *K*-value was measured, according to the method described by Choi et al. [72] and by using high-performance liquid chromatography (HPLC) (1100 series; Agilent Technologies, Palo Alto, CA, USA). The nucleic acid-related compounds (NARCs) (adenosine triphosphate (*ATP*), adenosine diphosphate (*ADP*), adenosine monophosphate (*AMP*), inosine monophosphate (*IMP*), inosine, (*HXR*), and hypoxanthine (*HX*)) were analyzed under the following conditions: UV detection at 254 nm, the absorbed dose range (AUF) was 0.5, µBondapak column C18 (3.9 mm × 300 mm; water, Milford, MA, USA), and the column oven temperature was 40 °C, the flow rate was 2.0 mL/min, and the mobile phase was 1% triethylamine (pH 6.5) modified with 10% H_3_PO_4_. The K-value was calculated using the following equation:

(2) $$K-value\;\left(\%\right)=\frac{\left[\left(HXR\right)+\left(HX\right)\right]}{\left[\left(ATP\right)+\left(ADP\right)+\left(AMP\right)+\left(IMP\right)+\left(HXR\right)+\left(HX\right)\right]}\times 100$$

### 4.7. Microbiological Analysis

The total viable count (TVC) and psychotropic count (PTC) as well as yeasts and molds were determined by the method described by Yu et al. [44], under aseptic conditions 10 g of each sample was naturalized with 90 mL of sterile normal saline (0.85%). A series of dilutions were then prepared from each sample and an aliquot (1 mL) of the diluent was poured into a petri dish and mixed with platelet agar medium. The inoculated plates were incubated at 30 °C for 2 days to measure TVC. The inoculated plates were incubated at 10 °C for a week for PTC. The inoculated plates were incubated at 25 °C for 5 days to count the yeasts and molds. Using the overlay casting method using violet red bile glucose agar (VRBGA), Enterobacteriaceae were enumerated as the corresponding plates were incubated for 24 h at 37 °C [73].

*Escherichia coli* O157:H7 were enumerated using sorbitol-McConkey agar (SMAC). Dilutions were coated on SMAC using the casting plate technique and then the plates were incubated for 24 h at 37 °C [74].

According to the procedure described by Tang et al. [75] *Pseudomonas fluorescens* were enumerated on king agar medium, and the inoculated plates were then incubated for 24 h at 30 °C.

Using de man rogosa Sharpe agar (MRS) and under anaerobic conditions lactic acid bacteria (LAB) were enumerated at 30 °C for 24 h [76].

All counts were expressed as log10 colony-forming units (CFU) g^−1^.

### 4.8. Sensory Evaluation

A sensory evaluation of cooked mackerel fillet samples (microwave oven for six min at 60% of maximum power (Mw60)) was conducted by 25 trained panelists of staff members (aged 21–40 years) of the Department of Food Science and Technology, Faculty of Agriculture, Menofiya University, according to the method described by Allam et al. [74,77]. Panelists were selected based on their interests and availability. The panelists were asked to rate the cooked samples’ color and odor using a scale point ranging from 0 to 10, where 10 = excellent; 9 = very good; 8 = good; 7 = acceptable; 6 = poor. The product was defined as unacceptable after the onset of a bad odor or unpleasant taste. The fresh mackerel fillet was used as a reference. The sensory analysis was done in three independent sessions.

### 4.9. Statistical Analysis

The study was replicated three times. Data were analyzed using the SPSS software (IBM SPSS statistics 21). Mean values of different parameters were used to compare chemical and microbiological indices. Sensory attributes data were analyzed by analysis of variance (ANOVA). The mean values ± standard deviation (SD) of the analyses were calculated. When a significant main effect was detected, the means were separated with the least significant difference (LSD) procedure. A two-way analysis of variance was used for multiple variable comparisons. Using analysis of variance (ANOVA), Tukey’s test, and independent sample *t*-test for all data interpretation at *p* < 0.05.

## Figures and Tables

**Table 1:** Test probabilities for physicochemical, microbiological, and sensory quality indices of mackerel tuna fillets—multi-aspect variance analysis, including interactions.

Quality Indices		Effect		Interaction T × ST
		Treatment (T)	Storage Time (ST)	
Physicochemical properties	pH	XXX ^1^	XXX	XXX
	Peroxide value	XXX	XXX	XXX
	TBARS	XX ^2^	XXX	XXX
	TVB-N	XXX	XXX	XXX
	K-value	X ^3^	XXX	XXX
Microbiological analyses	TVC	XX	XXX	XXX
	PTC	XX	XXX	XXX
	Enterobacteriaceae	XX	XXX	XXX
	*E*. *coli*	XXX	XXX	XXX
	*Pseudomonas fluorescens*	XXX	XXX	XXX
	Lactic acid bacteria	XXX	XXX	XXX
	Yeasts/molds	XXX	XXX	XXX
Sensory evaluation	Taste	XXX	XX	XXX
	Odor	XXX	XXX	XXX
	Overall acceptability	XXX	XXX	XXX

^1^ XXX: significant effect (*p* < 0.001); ^2^ XX: significant effect (*p* < 0.01); ^3^ X: significant effect (*p* < 0.05).

## Data Availability

The data presented in this study are available on request from the corresponding author.

## Supplementary Materials

The following supporting information can be downloaded at: www.mdpi.com/article/10.3390/gels8070405/s1, Table S1: Effect of storage conditions (2 °C for 20 days) and treatments on sensory evaluation during storage of mackerel tuna fillets.
