# Supplementary material for: Edible Xanthan/Propolis Coating and Its Effect on Physicochemical, Microbial, and Sensory Quality Indices in Mackerel Tuna (Euthynnus affinis) Fillets during Chilled Storage
Source: Gels. 2022 Jun 25;8(7):405. doi: 10.3390/gels8070405 (PMC9315731; doi:10.3390/gels8070405)
Supplement: Supplementary file 1 [file gels-08-00405-s001.zip › gels-1733246-supplementary.pdf]

gels

MDPI

Article

Edible Xanthan/Propolis Coating and Its Effect on Physicochemical, Microbial, and Sensory Quality Indices in Mackerel Tuna (*Euthynnus affinis*) Fillets during Chilled Storage

Aly Farag El Sheikha [1,2,3,4,5,*,†], Ayman Younes Allam [5,†], Emel Oz [6], Muhammad Rizwan Khan [7], Charalampos Proestos [8,*] and Fatih Oz [6]

1 College of Bioscience and Bioengineering, Jiangxi Agricultural University, 1101 Zhimin Road, Nanchang 330045, China
2 School of Nutrition Sciences, Faculty of Health Sciences, University of Ottawa, 25 University Private, Ottawa, ON K1N 6N5, Canada
3 Bioengineering and Technological Research Centre for Edible and Medicinal Fungi, Jiangxi Agricultural University, 1101 Zhimin Road, Nanchang 330045, China
4 Jiangxi Key Laboratory for Conservation and Utilization of Fungal Resources, Jiangxi Agricultural University, 1101 Zhimin Road, Nanchang 330045, China
5 Department of Food Science and Technology, Faculty of Agriculture, Minufiya University, Shibin El Kom 32511, Egypt; ayman_alaam@yahoo.com
6 Department of Food Engineering, Faculty of Agriculture, Ataturk University, Erzurum 25240, Turkey; emel.oz@atauni.edu.tr (E.O.); fatihoz@atauni.edu.tr (F.O.)
7 Department of Chemistry, College of Science, King Saud University, Riyadh 11451, Saudi Arabia; mrkhan@ksu.edu.sa
8 Laboratory of Food Chemistry, Department of Chemistry, School of Sciences, National and Kapodistrian University of Athens, 15772 Athens, Greece
* Correspondence: elsheikha_aly@yahoo.com (A.F.E.S.); harpro@chem.uoa.gr (C.P.)
† These authors are considered the first author.

Supplementary Materials

Table S1. Effect of storage conditions (2 °C for 20 days) and treatments on sensory evaluation during storage of mackerel tuna fillets.[1.]

Sensory attribute	Treatment [2]	Storage period (day)[1]					LSD
		0	5	10	15	20	
Odor	**Control**	9.6 ± 0.15 Aa	6.9 ± 0.13 Bb	5.2 ± 0.11 Cc	3.5 ± 0.14 Dc	2.5 ± 0.14 Ec	0.78
	XAN-EEP 0%	9.6 ± 0.12 Aa	7.8 ± 0.13 Bb	6.2 ± 0.14 Cb	4.5 ± 0.13 Db	2.7 ± 0.15 Ec	0.67
	XAN-EEP 1%	9.5 ± 0.09 Aa	7.7 ± 0.15 Bb	6.4 ± 0.18 Cb	4.6 ± 0.11 Db	3.8 ± 0.22 Eb	0.38
	XAN-EEP 2%	9.5 ± 0.15 Aa	8.9 ± 0.12 Ba	7.6 ± 0.08 Ca	6.5 ± 0.5 Da	5.6 ± 0.25 Ea	0.35
LSD		0.46	0.12	0.44	0.21	0.44	-
Test	Control	9.50 ± 0.31 Aa	7.21 ± 0.11 Bb	5.53 ± 0.15 Cb	4.11 ± 0.14Dd	2.4 ± 0.32 Dd	0.55
	XAN-EEP 0%	9.49 ± 0.29 Aa	8.18 ± 0.12 Ab	6.25 ± 0.18 Bb	5.15 ± 0.12 Cc	3.84 ± 0.29 Dc	0.90
	XAN-EEP 1%	9.49 ± 0.25 Aa	8.61 ± 0.15 Aa	6.56 ± 0.22 Bb	5.81 ± 0.15 Cb	4.42 ± 0.28 Db	0.83
	XAN-EEP 2%	9.50 ± 0.24 Aa	8.72 ± 0.24 Aa	7.84 ± 0.17 Ba	6.72 ± 0.19 Ca	5.62 ± 0.22 Da	0.54
LSD		0.23	0.30	0.42	0.22	0.41	-
Overall acceptability	Control	9.1 ± 0.35 Aa	6.3 ± 0.31 Bc	5.0 ± 0.25 Cc	3.2 ± 0.33 Dc	2.00 ± 0.29 Ec	1.08
	XAN-EEP 0%	9.2 ± 0.32 Aa	7.5 ± 0.34 Bb	6.11 ± 0.26 Cb	4.2 ± 0.41 Db	2.2 ± 0.32 Ec	1.11
	XAN-EEP 1%	9.2 ± 0.39 Aa	7.6 ± 0.29 Bb	6.13 ± 0.22 Cb	4.1 ± 0.38 Db	3.2 ± 0.41 Eb	1.21
	XAN-EEP 2%	9.2 ± 0.36 Aa	8.3 ± 0.36 Ba	7.2 ± 0.28 Ca	6.0 ± 0.39 Da	4.5 ± 0.40 Ea	1.30
LSD		0.22	0.23	0.28	0.19	0.35	-

[1] Results are expressed as Means ± standard deviation (SD). Values with different alphabetical letters (small letters, a to d) within columns differ significantly ($P < 0.05$) as "effect of treatment". Values with different alphabetical letters (capital letters, A to E) within rows differ significantly ($P < 0.05$) as "effect of storage period". All the readings were taken in triplicates.

[2] Treatment: Control: Uncoated mackerel tuna fillet samples; XAN-EEP 0%: Coated samples with xanthan containing (0%) ethanolic extract of propolis; XAN-EEP 1%: Coated sample with xanthan containing (1%) ethanolic extract of propolis; XAN-EEP 2%: Coated sample with xanthan containing (2%) ethanolic extract of propolis.
